# Isolation and Molecular Characterization of Free-Living Amoebae from Different Water Sources in Italy

**DOI:** 10.3390/ijerph120403417

**Published:** 2015-03-24

**Authors:** Margherita Montalbano Di Filippo, Maristella Santoro, Piero Lovreglio, Rosa Monno, Carmen Capolongo, Carla Calia, Luciana Fumarola, Rossella D’Alfonso, Federica Berrilli, David Di Cave

**Affiliations:** 1 Department of Experimental Medicine and Surgery, University of Rome Tor Vergata, Via Montpellier 1, 00133 Rome, Italy; E-Mails: montalbano.margherita89@gmail.com (M.M.F.); maristella5384@gmail.com (M.S.); dicave@uniroma2.it (D.C.); 2 Interdisciplinary Department of Medicine, University of Bari, Piazza G. Cesare 11, 70124 Bari, Italy; E-Mail: piero.lovreglio@uniba.it; 3 Department of Basic Medical Science, Neuroscience and Sense Organ, University of Bari, Piazza G. Cesare 11, 70124 Bari, Italy; E-Mails: rosa.monno@uniba.it (R.M.); carmen.capolongo84@gmail.com (C.C.); carla.calia@virgilio.it (C.Cal.); luciana.fumarola@uniba.it (L.F.); 4 Department of Systems Medicine, University of Rome Tor Vergata, Via Montpellier 1, 00133 Rome, Italy; E-Mail: dalfonso@uniroma2.it; 5 Laboratory of Parasitology, Foundation Polyclinic Tor Vergata, Viale Oxford 81, 00133 Rome, Italy

**Keywords:** free-living amoebae, molecular characterization, water sources, Italy

## Abstract

Free-living amoebae (FLA) are protozoa ubiquitous in Nature, isolated from a variety of environments worldwide. In addition to their natural distribution, some species have been found to be pathogenic to humans. In the present study a survey was conducted in order to evaluate the presence and to characterize at molecular level the isolates of amoebic organisms collected from different water sources in Italy. A total of 160 water samples were analyzed by culture and microscopic examination. FLA were found in 46 (28.7%) of the investigated water samples. Groundwater, well waters, and ornamental fountain waters were the sources with higher prevalence rates (85.7%, 50.0%, and 45.9%, respectively). Identification of FLA species/genotypes, based on the 18S rDNA regions, allowed to identify 18 (39.1%) *Acanthamoeba* isolates (genotypes T4 and T15) and 21 (45.6%) *Vermamoeba vermiformis* isolates. Other FLA species, including *Vahlkampfia* sp. and *Naegleria* spp., previously reported in Italy, were not recovered. The occurrence of potentially pathogenic free-living amoebae in habitats related to human population, as reported in the present study, supports the relevance of FLA as a potential health threat to humans.

## 1. Introduction

Free-living amoebae (FLA) are widespread protozoa not requiring a host organism for survival, having their natural habitats in the environment (e.g., soil, air and a multiplicity of aquatic environments). Some of them may occasionally infect and cause diseases in humans and other animals [1]. Currently, the causative agents of diseases in humans are classified as belonging to two super-groups: Amebozoa, including the genera *Acanthamoeba*, *Balamuthia*, *Sappinia* and *Hartmannella*, and Excavata, including the genus *Naegleria* [2,3]. In particular, *Naegleria fowleri* is responsible for primary amoebic meningoencephalitis (PAM), while some species of *Acanthamoeba* and *Balamuthia mandrillaris* induce granulomatous amoebic encephalitis (GAE), mainly in immunocompromised patients. Also, *Acanthamoeba* species may give rise to a severe corneal infections designated as amoebic keratitis (AK). The phylogenetic relationships within the genus *Acanthamoeba* are far from being fully resolved. To date, on the basis of rDNA sequences, the genus has been divided into 20 genotypes (T1–T20) [4]. To date, several genotypes are described as the causative agent of different diseases, the genotypes T4, T3, and T11 are the most often associated with keratitis [5,6]. Moreover, the species *Hartmannella vermiformis,* rarely associated with AK [7,8], has been renamed as *Vermamoeba vermiformis,* because its significant differentiation from all other *Hartmannella* species [2]. Studies on the epidemiology of free-living pathogenic amoebae have been conducted all over the world but their diffusion in the environment in Italy is still poorly understood. The first report about the occurrence of FLA is referred to the study by Scaglia *et al.* [9], reporting the detection of *Naegleria australiensis* in northern Italy. Some years later, the same authors also reported the presence of *Naegleria* spp., *Acanthamoeba* spp., *Vahlkampfia* spp., and *Hartmannella* spp., in samples from thermal areas [10]. Recently, Corsaro and Venditti [11] conducted a molecular study signaling the presence of the new *Acanthamoeba* T16 genotype in southern Italy.

The aims of the present study were to identify FLA from water sources in Italy and to characterize the isolates at species/genotypes level to better understand their environmental distribution and to evaluate the potential risk to human health.

## 2. Materials and Methods

### 2.1. Sample Collection and Culture of FLA

**Figure 1:**
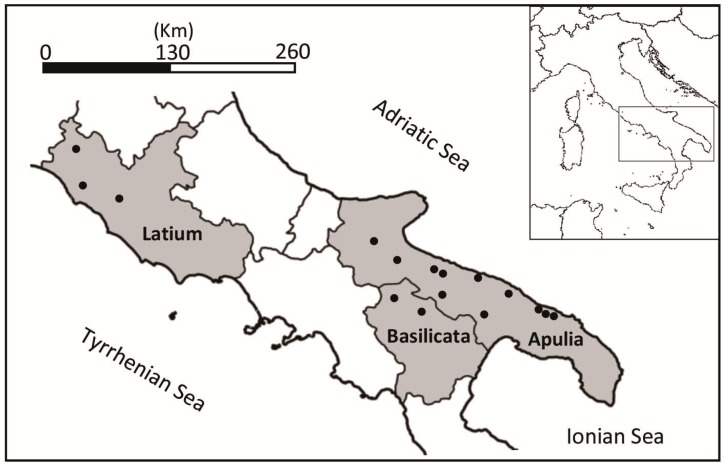
Studied area and water sampling sites (dots).

A total of 160 water samples were collected in the central (Latium) and southern (Apulia and Basilicata) regions of Italy between 2007 and 2013 (Figure 1).

All samples were classified in relation to the different water sources as reported in Table 1.

**Table 1:** Water isolation sources, number of samples examined and results obtained by culture and molecular analysis of free-living amoebae in Italy.

Water Samples *	Samples Examined	Cultured Positive Samples	Molecular Identification	
	Total	Number (%)	*Acanthamoeba* (Genotype)	*V. vermiformis*
Well water (W)	4	2 (50.0)	2 (T15)	-
Thermal water (T)	9	3 (33.3)	2 (T4)	-
Tap water (TW)	84	15 (17.8)	4 (T4)	8
Bottled mineral water (M)	19	3 (15.8)	-	3
Ornamental fountain water (F)	37	17 (45.9)	6 (T4)	10
Groundwater (G)	7	6 (85.7)	1 (T4); 3 (T15)	-
Total	160	46 (28.7)	18 (13 T4; 5 T15)	21

* Water temperatures were always below 25 °C.

From each sampling focus, a total of 1 liter of water was collected in a sterile plastic bottle and immediately transported at ambient temperature to the laboratory. Water samples were filtered by a vacuum filtration system with a Millipore 0.45 μm pore size filter (Merck Millipore, Darmstadt, Germany). The membrane filters were minced in 10 mL of sterile phosphate-buffered saline pH 7.2 (PBS), homogenized by vortex for 5 min and centrifuged at 1200 g for 10 min. 200 μL of pellet were inoculated on non-nutrient agar (NNA) with a lawn of heat inactivated *Escherichia coli* in Page’s Amoeba Saline solution (PAS), and incubated at 30 °C. The plates were observed daily for amoebic growth up to 21 days after inoculation by inverted microscope at 200× and 400× magnification.

### 2.2. Molecular Methods

From all positive samples, the growing amoebae were harvested from culture plates, placed in Eppendorf tubes and washed two times with PBS, pH 7.4, before molecular procedure. DNA extraction was performed by using the QIAamp DNA Micro Kit (Qiagen, Milan, Italy). For identification of *Acanthamoeba*, a PCR was carried out to amplify a 18S rDNA region defined as ASA.S1 that includes the hipervariable diagnostic fragment 3 (DF3), using the genus-specific primers JDP1 and JDP2 [12], and subsequent comparison of the obtained sequences with types T1–T20 available from GeneBank. To identify other FLA species, the 18S rDNA amplification with primers P-FLA-F and P-FLA-R was performed. This PCR allows the simultaneous amplification of FLA, amplicons lengths varying from 500 to 1500 base pairs (*V. vermiformis*: 800 bp; *N. fowleri*: 900 bp; *Vannella* sp., *Vahlkampfia ovis*: 950bp; *Acanthamoeba* species: from 1080 to 1500 bp) as described previously [13]. All PCRs were carried out in a 25 μL volume containing 12.5 μL PCR master mix 2X (Promega, Milan, Italy), 5μL template DNA, and 0.6 mM of each primer and were performed in a TProfessional Basic Thermocycler (Biometra GmbH, Göttingen, Germany).The PCR products were visualized by electrophoresis on 1% agarose gel stained by SYBR Safe DNA gel stain (Invitrogen, Monza MB, Italy). PCR amplicons were purified using the mi-PCR Purification Kit (Metabion GmbH, Steinkirchen, Germany) according to the manufacturer’s instructions and directly sequenced on both strands by the Bio-Fab Research (Rome, Italy). Sequences were edited with the FinchTV 1.4 software (Geospiza, Inc, Seattle, WA, USA) and aligned by ClustalW2 and. To assign the isolates to the specie/genotype level, phylogenetic analysis was performed by comparison of the obtained sequences with those of reference strains. A Neighbor joining phylogenetic trees were constructed with MEGA version 5 [14] and statistical reliability of nodes was evaluated using 1000 pseudoreplication bootstrap. The best model of tree construction was selected using JModelTest by the Akaike Information Criterion (AIC) [15]. Sequences generated in this study were deposited in the GenBank database and are available under the accession numbers: KP756942-KP756959 and KP792377-KP792398.

## 3. Results

### 3.1. Microscopy

Overall, 46 water samples out of 160 (28.7%) were positive in the culture examination for the presence of amoebic cells. Groundwater (G), well waters (W) and ornamental fountain waters (F), were the sources with the highest number of positive samples, showing 6/7 (85.7%), 2/4 (50.0%), and 17/37 (45.9%) of culture-positive isolates, respectively. A lower rate of positivity was obtained from thermal water (T) (33.3%), tap water (TW) (17.8%) and bottled mineral water (M) (15.8%). Sources and numbers of positive samples are shown in Table 1.

### 3.2. Molecular Identification and Genotyping

**Figure 2:**
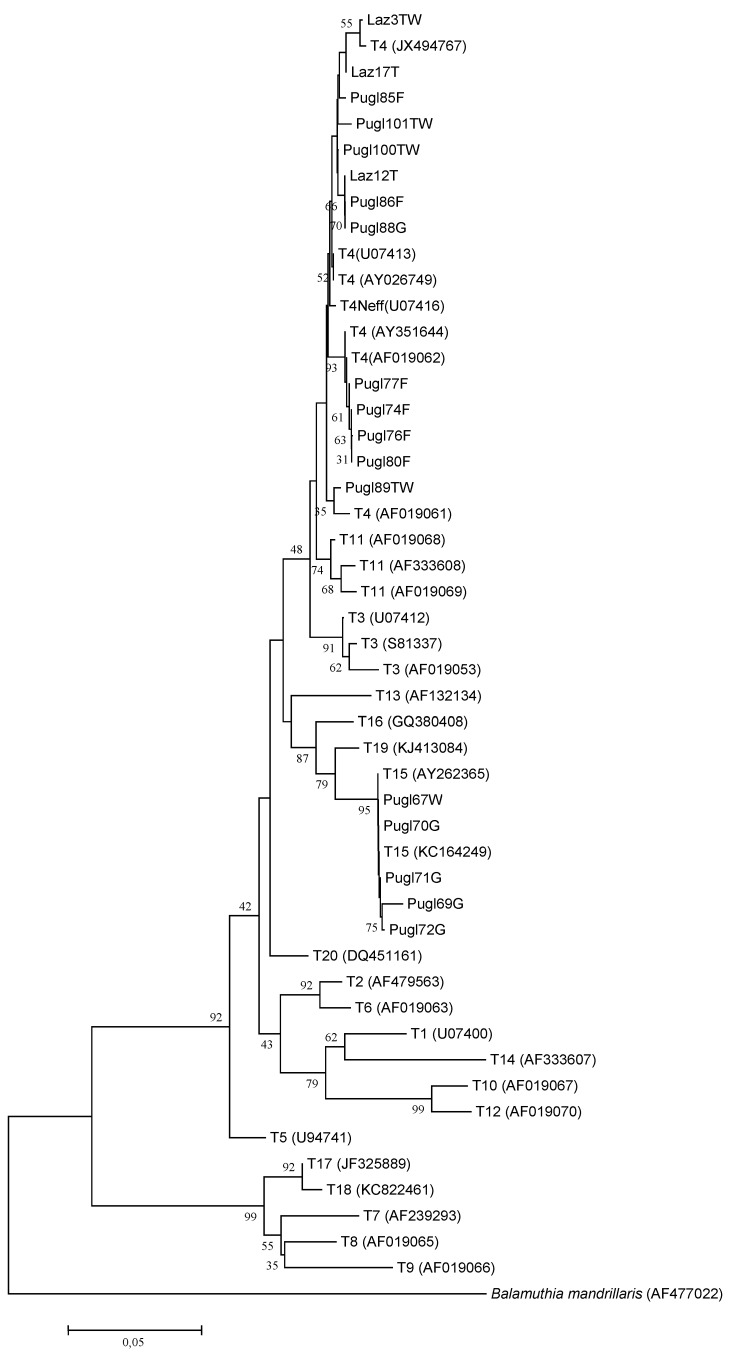
Phylogenetic relationships of 18 *Acanthamoeba* isolates inferred by NJ analysis based 18S-rRNA gene sequences. Only bootstrap values >35 are reported. GenBank accession numbers of reference strains are indicated in parenthesis.

Eighteen out of 46 (39.1%) samples from all water sources except bottled mineral water, resulted positive to *Acanthamoeba* after JDP1/JDP2 selective amplification. The phylogenetic analysis carried out by the comparative investigation of the sequences obtained in this study with all the representative *Acanthamoeba* strains available from GenBank identifies two well defined clusters corresponding to *Acanthamoeba* genotypes T4 and T15 (Figure 2). The potential pathogenic *Acanthamoeba* T4 was the most common genotype detected in 13/18 isolates (72.2%) distributed in all sampling sites while 5/18 (27.7%) samples (1 well water and 4 groundwater) from Basilicata (n = 3) and from Apulia (n = 2), were assigned to T15 genotype (Figure 2).

**Figure 3:**
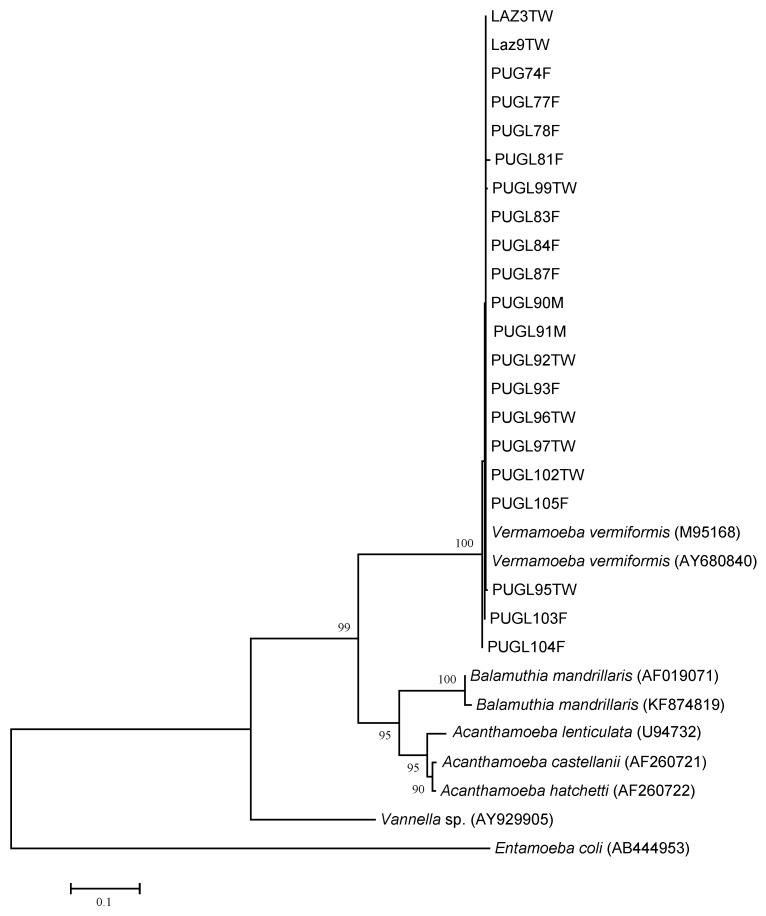
Phylogenetic relationships of 21 FLA isolates inferred by NJ analysis based on 18S ribosomal DNA sequences. Only bootstrap values >70 are reported. GenBank accession numbers of reference strains are indicated in parenthesis.

Among the 46 isolates microscopically positive to the culture examination, 42 provided PCR amplification with primers P-FLA-F and P-FLA-R, while four samples yielded no positive signals. Eighteen isolates showed bands with approximate sizes of ~1100 bp (expected for *Acanthamoeba* spp.), consistently with the sample identification obtained with JDP1/JDP2 analysis. Thirty-three/42 PCR positive isolates showed bands with approximate sizes of ~800 bp (expected for *V. vermiformis*). DNA sequencing of eight positive amplicons was unsuccessful due to insufficient and/or ineffective templates while non-interpretable sequences were obtained from three of the amplified samples. To assign the remaining 22 isolates to the specie level, a comparison with available sequences in GenBank was performed by BLAST. The analyses unambiguously identified 21 samples as *V. vermiformis* (45.6%)*,* the sequences showing the highest identity (99%) with those accessible in GenBank. The isolate Pugl67W (KP792398) gave 99% similarity with DNA sequences from *Amoebozoa* sp. amMP3 (JX312795) isolate, originated from a mud pond in South Italy and recently described [16]. Regarding the samples Laz3TW, Pugl74F and Pugl77F both bands of the above sizes were observed and sequenced, evidencing the simultaneous presence of *Acanthamoeba* spp. and *V. vermiformis*. No water sample analyzed in this study was characterized for the presence of other FLA species, including *Naegleria* spp. As regard *V. vermiformis* sequences, a phylogenetic analysis was also performed. A well-defined cluster corresponding to *V. vermiformis* species, supported by significant bootstrap values (100%) was evidenced in the NJ tree (Figure 3), thus confirming species assignment obtained by BLAST analysis.

## 4. Discussion

Because of their ubiquity in the environment, FLA have been frequently recovered in soil, air, and in water samples (fresh water lakes, natural thermal water, swimming pools) worldwide. Recently, in the study by Coşkun *et al.* [17] FLA were detected in 33 (22%) out of 150 water samples from various districts in Turkey, allowing the identification of two species of *Acanthamoeba* (*A. castellanii* and *A. polyphaga*) and *Vermamoeba (Hartmannella) vermiformis*. Similar results are described by Tsvetkova *et al.* [13] in a survey carried out in Bulgaria to determine the presence of free-living amoebae from environmental sources where FLA (identified as *Acanthamoeba* spp. and *Hartmannella* spp.) were recovered in 171 (61.1%) out of 280 samples. *Naegleria* spp. and *Vanella* spp., along with *Acanthamoeba* spp. and *H. vermiformis,* have been also identified in 64/83 culture positive (77.1%) water samples from treatment plants for the supply of drinking water in Spain [18]. Another study conducted in Spain in samples from different water matrices, showed 99.1% positivity for *Acanthamoeba* genotype T4 and the first report of *Balamuthia mandrillaris* in water, detected in a single sample [19]. Overall, the high variability observed in the prevalence values worldwide could be attributed to several factors, including the different applied methodologies, as well the features of the water source and of the geographical areas. However, the correct understanding of the factors influencing the occurrence of the different species appears of great concern, as these amoebae are free-living organisms, and their potential capabilities to cause severe infections of the central nervous system, ocular keratitis and other disorders are now ascertained worldwide [20,21]. Moreover, most of these protozoa may serve as reservoirs or/and vectors of a large number of intracellular pathogenic bacteria (such as *Legionella pneumophila*, *Pseudomonas* spp. and *Mycobacterium* spp.), and viruses [22,23]. Consequently, over the past 10 years, the association of pathogens with free-living amoebae gained increasing attention in relation to the risk to public health [24].

In Italy, only two epidemiological surveys on environmental samples were previously carried out. Pathogenic *Naegleria australiensis* was isolated from 7/30 (23.3%) therapeutic swimming pools and thermal mud basins of a spa in northern Italy. Later, the presence of *Naegleria* spp. (6%), *Acanthamoeba* spp. (5.2%), *Vahlkampfia* spp. (33.6%), and *Hartmannella* spp. (24.1%) was assessed in samples from 34 thermal baths and mud-basins in the same area [9,10]. In both studies, isolation was by culture. Despite these data, only a molecular study, where the new *Acanthamoeba* genotype T16 in a freshwater pond was described, is so far available in Italy [11]. In the present study, FLA were isolated in all water samples analyzed (well water, thermal water, tap water, bottled mineral water, ornamental fountain water, and groundwater). Our results evidenced a wide distribution in the environment of *Acanthamoeba* spp. (39.1%) and *V. vermiformis* (45.6%) while no isolates of other FLA, including *Vahlkampfia* sp., and *Naegleria* spp. were detected, although previously reported in Italy. In northern Italy, the presence of *Naegleria fowleri* was also described in a case of postmortem diagnosed PAM in a child who probably acquired the infection after swimming in a river [25]. The absence of other species in our samples could be due to different issues: the lower prevalence in the environment; the ecological characteristics of the collection sites, but also the different methodologies used for the identification (microscopic or molecular); finally, the cultural temperature (30 °C), not adequate for all the species, particularly for *Naegleria* spp. However, a wider distribution of *Acanthamoeba* and *Vermamoeba* in human-related habitats compared with other FLA has been described in other studies in Europe [13,17,26,27].

Based on the phylogenetic analysis, the potential pathogenic *Acanthamoeba* T4 was the primary genotype detected in water samples from all the studied area, while T15 genotype was observed only in samples from Apulia and Basilicata. According to many studies carried out so far, *Acanthamoeba* T4 genotype is the strain most commonly isolated in the environment worldwide and the most frequent genotype causing AK and other diseases in humans [5,6,28]. The genotype T15, described by Hewett *et al.* [29] and associated with the species *Acanthamoeba jacobsi*, has been initially isolated only from environmental sources. The first case of AK associated with genotype T15 was reported from Italy by Di Cave *et al.* [30]. In Italy, T4 and T15 genotypes related to *Acanthamoeba* keratitis have been previously described, representing the predominant *Acanthamoeba* genotypes involved in these serious infections (approximately 90% of cases) [30,31,32].

Noteworthy, in the present study, the detection of FLA by the growth of amoebae in culture and not only by PCR methods indicates their viability. The high resistance of FLA cysts to the water treatments and the ability of trophozoites to multiply in water networks may determine the wide spread of potential pathogenic free-living amoebae in habitats related to human population [33].

## 5. Conclusions

The present study has contributed to add new molecular data on free-living amoebae in Italy. Considering the presence of potentially pathogenic species in different water sources, these findings also showed the need of further epidemiological studies for a better understanding of the role of FLA as a potential health threat to humans, and to know possible sources of infections caused by these microorganisms.
